# Adsorption and Desorption Properties of Polyethylenimine/Polyvinyl Chloride Cross-Linked Fiber for the Treatment of Azo Dye Reactive Yellow 2

**DOI:** 10.3390/molecules26061519

**Published:** 2021-03-10

**Authors:** Zhuo Wang, Ha Neul Park, Sung Wook Won

**Affiliations:** 1 Department of Ocean System Engineering, College of Marine Science, Gyeongsang National University, Tongyeong 53064, Korea; wz928661411@gmail.com (Z.W.); parksky@gnu.ac.kr (H.N.P.); 2 Department of Marine Environmental Engineering, College of Marine Science, Gyeongsang National University, Tongyeong 53064, Korea

**Keywords:** reactive dyes, polyethylenimine, polyvinyl chloride, adsorption, reusability

## Abstract

In this study, the optimal conditions for the fabrication of polyethylenimine/polyvinyl chloride cross-linked fiber (PEI/PVC-CF) were determined by comparing the adsorption capacity of synthesized PEI/PVC-CFs for Reactive Yellow 2 (RY2). The PEI/PVC-CF prepared through the optimal conditions was characterized using scanning electron microscopy (SEM), Fourier transform infrared spectroscopy (FTIR), and Brunauer–Emmett–Teller (BET) analyses. Several batch adsorption and desorption experiments were carried out to evaluate the sorption performance and reusability of PEI/PVC-CF for RY2. As a result, the adsorption of RY2 by PEI/PVC-CF was most effective at pH 2.0. A pseudo-second-order model fit better with the kinetics adsorption data. The adsorption isotherm process was described well by the Langmuir model, and the maximum dye uptake was predicted to be 820.6 mg/g at pH 2.0 and 25 °C. Thermodynamic analysis showed that the adsorption process was spontaneous and endothermic. In addition, 1.0 M NaHCO_3_ was an efficient eluent for the regeneration of RY2-loaded PEI/PVC-CF. Finally, the repeated adsorption–desorption experiments showed that the PEI/PVC-CF remained at high adsorption and desorption efficiencies for RY2, even in 17 cycles.

## 1. Introduction

The consumption of synthetic dyes has grown with the rapid development of human society. It is estimated that more than 10,000 kinds of dyes are used in industries and the annual output of dyes exceeds 700,000 tons worldwide [1]. The most widely used dyes are azo dyes, accounting for about 70% of reactive dye consumption [2]. However, up to 50% of azo dyes remain in the used dye bath, and they can discharge into the environment without appropriate treatment [3]. Azo dyes are resistant to physical and chemical oxidizing agents, making it difficult to treat azo dyes containing wastewater with conventional physical and chemical methods [4]. The direct emission of azo dye wastewater into the environment is a big threat to human beings as well as the ecosystem. Many azo dyes and their degradation products are toxic, mutagenic, and carcinogenic to humans [5]. The existence of dyes in the aquatic environment can prohibit the penetration of sunlight into the water, which influences the photosynthesis activities of aquatic plants, and eventually damages the whole aquatic ecosystem [6]. Therefore, it is of great significance to remove dyes from the dye-baring wastewater before discharging them into the environment.

Various kinds of physical, chemical, and biological methods, such as coagulation/flocculation, adsorption, membrane filtration, chemical oxidation, electrocatalytic degradation, and biodegradation, have been applied for dye wastewater treatment [7,8]. Despite their advantages, there are also many significant disadvantages such as low efficiency, secondary pollution, generation of toxic byproducts and sludge, and high operating costs [9]. On the other hand, adsorption is known to be one of the most promising methods to remove dyes from aqueous solutions, owing to its high efficiency, low cost, and easy operation [10]. Various kinds of sorbents, including activated carbon, ion-exchange resin, algae, nanocomposite, etc., have been used to remove dyes from wastewater [11,12]. Nevertheless, these adsorbents showed some drawbacks, such as low adsorption capacity, separation, and regeneration difficulties [13,14]. Therefore, it is very important to develop adsorbents with high adsorption capacity and easy separation and regeneration, which can open up cost-effective opportunities for the treatment of dye-bearing wastewaters.

Polyvinyl chloride (PVC) is widely used in daily life, and a large number of PVC products have become plastic waste. Recycling PVC is challenging because of the cross-contamination with polyethylene terephthalates and generation of toxic compounds during the pyrolysis process; therefore, most of the PVC waste ends up in landfills [15,16,17]. Establishing a sustainable approach to the recycling of waste PVC is of great interest to the environmental communities. For example, a convenient route has been developed to bond amines and alkyl chloride groups by nucleophilic substitution reactions through secondary chlorine groups in the PVC backbone [18]. Polyethylenimine (PEI) is an amine-rich water-soluble polymer that is widely used for surface modification [19]. Therefore, PVC can be modified by PEI through the nucleophilic substitution reaction.

In this study, PVC and PEI were simply cross-linked and then extruded to make fibrous polyethylenimine/polyvinyl chloride cross-linked fiber (PEI/PVC-CF) adsorbents, which have a high adsorption capacity, low cost, and are easy to separate and reuse. Through a series of experiments considering the mass ratio of PEI and PVC and the cross-linking time, and PEI/PVC-CF with an excellent adsorption performance for Reactive Yellow 2 (RY2) was implemented. The adsorption properties of PEI/PVC-CF for RY2 were examined in various batch adsorption experiments. In addition, the eluents for dye desorption were investigated and the desorption properties and reusability of the developed adsorbent were confirmed by repeated adsorption–desorption experiments.

## 2. Results and Discussion

### 2.1. The Influences of Mass Ratio and Cross-Linking Time

**Figure 1:**
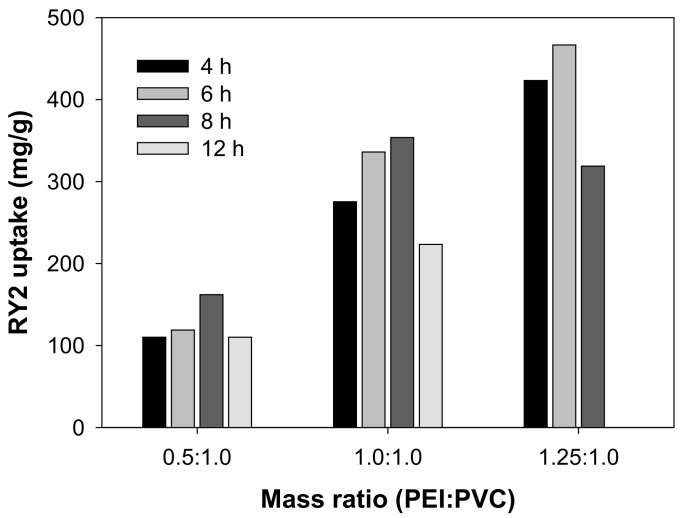
Comparison of Reactive Yellow 2 (RY2) uptake on polyethylenimine/polyvinyl chloride cross-linked fiber (PEI/PVC-CF) prepared under different conditions (Mass ratio (PEI:PVC) = 0.5:1.0, 1.0:1.0, and 1.25:1.0; cross-linking time = 4, 6, 8, and 12 h; adsorption conditions = *C_i_* 500 mg/L, pH 2.0, 24 h, and 25 °C).

A variety of PEI/PVC-CFs fabricated at different mass ratios (PEI:PVC) and cross-linking times were evaluated through adsorption experiments in 500 mg/L of RY2 solution. As shown in Figure 1, the dye uptake varied sensitively depending on the change in the mass ratio and cross-linking time. The RY2 uptake on PEI/PVC-CFs was initially greatly influenced by the mass ratio of PEI and PVC, and the dye uptake increased from 110.14 to 423.26 mg/g when the PEI content ratio increased from 0.5 to 1.25 at the cross-linking time of 4 h. According to our previous report [20], PEI/PVC cross-linked polymers were synthesized because of the alkylation of PEI by PVC under the given experimental conditions. As a result, many free amine groups that can participate in binding to RY2 were introduced into the synthesized polymer. Thus, the increase in PEI content can be expected with more free amine groups. Cross-linking time served as a second important factor in determining the adsorption amount of the adsorbent. For all three different mass ratios, the RY2 adsorption amount initially tended to increase with increasing cross-linking time but decreased after a certain cross-linking time. It can be considered that cross-linking time affects the degree of alkylation between PEI and PVC, and eventually, excessive alkylation can reduce the number of amine groups, thereby reducing the adsorption performance of the adsorbent. The mass ratios of 0.5:1.0 and 1.0:1.0 (PEI:PVC) exhibited optimal adsorption capacities of 162.14 and 353.68 mg/g, respectively, at the cross-linking time of 8 h. On the other hand, in the case of the mass ratio of 1.25:1.0, the best adsorption capacity of 466.57 mg/g was obtained at the cross-linking time of 6 h. Therefore, the mass ratio of 1.25:1.0 (PEI:PVC) and the reaction time of 6 h at 80 °C were selected as the optimal manufacturing conditions for PEI/PVC-CF adsorbent.

### 2.2. Characterization of the Optimized PEI/PVC-CF

**Figure 2:**
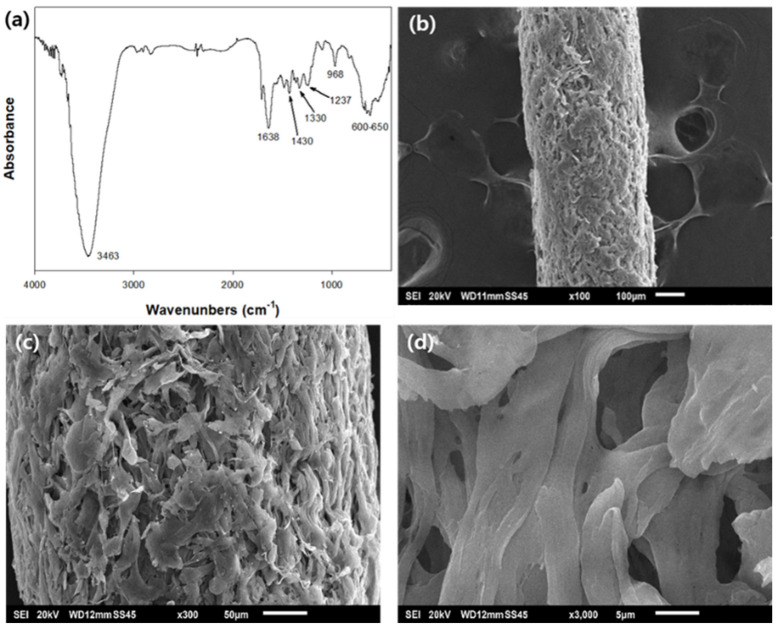
Fourier transform infrared spectroscopy (FTIR) spectrum of PEI/PVC-CF (**a**) and scanning electron microscopy (SEM) images of PEI/PVC-CF at 100× (**b**), 300× (**c**), and 3000× (**d**) magnifications. (SEI: Secondary Electron Image; the SEM images were recorded at 20 kV, the units in Figure (**b**–**d**) were 100, 50, and 5 µm, respectively).

The Fourier transform infrared spectroscopy (FTIR) spectrum of PEI/PVC-CF prepared under the optimal conditions was analyzed, and the characteristic peaks of PEI and PVC were observed, as shown in Figure 2a. The typical bands for PEI were seen at 3463 cm^−1^ (N–H asymmetric stretching of amines), 1638 cm^−1^ (-NH_2_ for amine I or -NH for amide II), and 1430cm^−1^ (-NH bending and deformation vibration of -NH_2_), respectively [21,22,23]. The characteristic peaks of PVC were observed at 1330 cm^−1^ (-CH_2_ deformation), 1237 cm^−1^ (C–C stretching), and 600–650 cm^−1^ (C–Cl stretching), separately [24,25].

**Figure 3:**
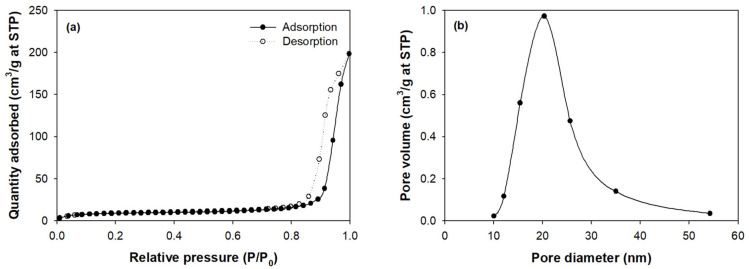
N_2_ adsorption–desorption isotherms (**a**) and pore size distribution curves (**b**) of PEI/PVC-CF. (STP: standard temperature and pressure).

Figure 2b–d show the SEM images of PEI/PVC-CF at 100×, 300×, and 3000× magnifications. As seen in Figure 2b, the surface of PEI/PVC-CF was quite rough and porous, and the adsorbent had a thickness of 400–420 μm. In Figure 2c, the PEI/PVC-CF was observed to have rich pore structures. In the image at 3000× magnification (Figure 2d), there were various irregular pores of several micrometers. To further investigate the pore structural properties of PEI/PVC-CF, the Brunauer–Emmett–Teller (BET) surface area analysis was carried out by N_2_ adsorption–desorption isotherms, and the surface area, pore volume, and pore diameter were analyzed, as given in Figure 3a,b. According to the N_2_ adsorption isotherm in Figure 3a, there was little adsorption when the relative pressure was low, but adsorption increased sharply as the saturation pressure approached. In particular, in the relative pressure range of 0.8–1.0, the adsorption–desorption isotherms showed two different paths. The shape of the adsorption–desorption isotherm belongs to the type IV hysteresis loop, as defined by IUPAC [26], which indicates the existence of mesopores. This fact was confirmed through the pore distribution of PEI/PVC-CF (Figure 3b). The size of the mesopores was distributed in the range of 10–50 nm, and most of them existed in the size of 20 nm. The BET surface area of PEI/PVC-CF was 31.71 m^2^/g, while the total pore volume was calculated as 0.005 cm^3^/g. Also, the average pore size of the adsorbent was evaluated to be 20.36 nm. In conclusion, this pore structure of PEI/PVC-CF can promote the penetration, diffusion, and adsorption of RY2 molecules into the adsorbent.

### 2.3. Effect of pH on Dye Adsorption

**Figure 4:**
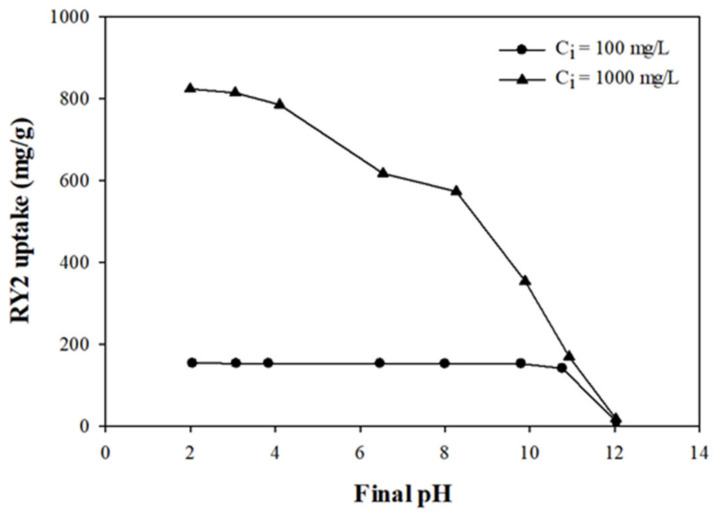
The effect of pH on RY2 adsorption by PEI/PVC-CF at the initial dye concentrations of 100 and 1000 mg/L (experimental conditions: *m* = 0.02 g, *V* = 30 mL, pH range = 2.0–12.0, and temperature = 25 °C).

The influence of pH on the adsorption properties of the optimized PEI/PVC-CF was investigated at the initial RY2 concentrations of 100 and 1000 mg/L, respectively, and the experimental results are shown in Figure 4. At an initial dye concentration of 100 mg/L, RY2 was completely removed over a broad pH range of 2.0 to 9.8, at which time the dye uptake was 153.6 mg/g. However, as the pH increased to 10.8, the adsorption amount decreased by about 8%, and it was hardly adsorbed at pH 12.0. As such, PEI/PVC-CF can be expected to effectively treat low concentration dyeing wastewater under acidic conditions as well as neutral or weak alkaline conditions. On the other hand, at a high concentration of 1000 mg/L, the effect of pH on dye adsorption by optimal PEI/PVC-CF was observed more clearly. The best dye uptake was found at pH 2.0, and as the pH increased from 2.0 to 4.0, the dye uptake decreased slightly by 5% from 823.3 to 784.5 mg/g. As the pH increased further to 6.5, the dye uptake dropped significantly to 616.9 mg/g, and it finally showed the worst adsorption amount at pH 12.0. Therefore, pH 2.0 was selected as the optimal pH for other adsorption studies.

This sorption tendency could be considered to be related to the p*K*_a_ values of the PEI molecule in the adsorbent. The branched PEI, consisting of primary (25%), secondary (50%), and tertiary (25%) amines, has three p*K*_a_ values of 4.5, 6.7, and 11.6 for 1°, 2°, and 3° amines, respectively [27]. Thus, below pH 5.0, all amine groups in PEI are fully protonated, so that positively charged amine groups can participate in binding with the negatively charged sulfonic groups of RY2 molecules by electrostatic attraction. However, if the pH of the dye solution increases to more than 5.0, the amine groups of PEI are gradually deprotonated, which is likely to result in a decrease in the amount of RY2 adsorption by PEI/PVC-CF. Similar results have been observed with recently reported adsorbents, such as PEI microgels for methyl orange, methylene blue, and PEI-chitin for Pd(II) [28,29].

### 2.4. Adsorption Kinetics

**Figure 5:**
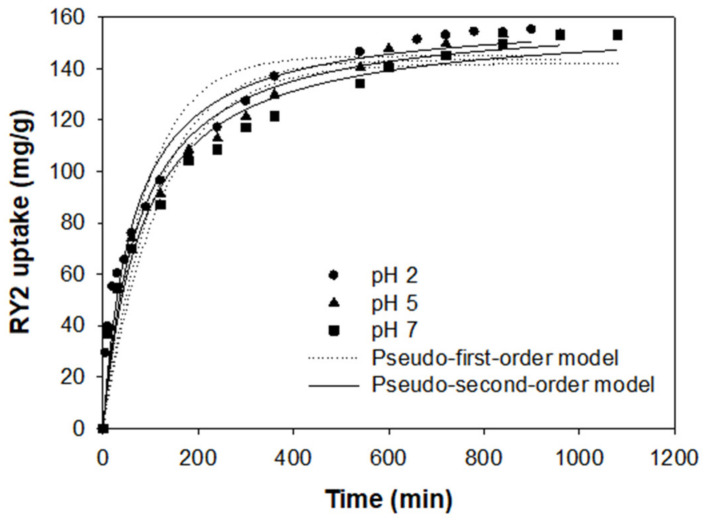
Kinetics of RY2 adsorption by PEI/PVC-CF at different pH values (Experimental conditions: *m* = 0.02 g, *V* = 30 mL, *C_i_* = 100 mg/L, and temperature = 25 °C).

Adsorption kinetics can provide vital information, such as equilibrium time, control mechanism, and adsorption rate, to select the best operation conditions for full-scale batch adsorption processes [30]. The kinetic experiments were evaluated at pH 2.0, 5.0, and 7.0, respectively, for an initial dye concentration of 100 mg/L using the optimized PEI/PVC-CF adsorbent. The experimental results are shown in Figure 5. In all pH conditions, the amount of adsorbed RY2 sharply increased at the initial stage and then gradually increased. Thereafter, at the adsorption time of 840 min or more, the adsorption equilibrium was reached, and the adsorption amount was almost constant. The dye uptake in the adsorption equilibrium was similar in all cases, but an apparent difference in dye uptake at different pHs was found before approaching adsorption equilibrium.

To better understand the adsorption kinetic process, the pseudo-first-order and pseudo-second-order models were used to describe the kinetic experimental data. The two non-linear kinetic models are expressed as follows in Equations (1) and (2):

Pseudo-first-order kinetic model:

(1) $$q_{t}=q_{1}\left(1-\exp\left(-k_{1}t\right)\right)$$

Pseudo-second-order kinetic model:

(2) $$q_{t}=\frac{q_{2}^{2}k_{2}t}{1+q_{2}k_{2}t}$$

where *q*_1_ and *q*_2_ are the amount of dye adsorbed at the equilibrium (mg/g), *q_t_* is the amount of dye at time *t* (mg/g), *k*_1_ is the pseudo-first-order rate constant (L/min), and *k*_2_ is the pseudo-second-order rate constant (g/mg min). In addition, the initial adsorption rate (*h*) at *t* → 0 can be defined by Equation (3):

(3) $$h=k_{2}q_{2}^{2}.$$

The kinetic models were calculated through non-linear regression analysis by SigmaPlot 10.0 software. The model parameters, *h,* the coefficient of determination (*R*^2^) values, and the mean percentage errors (*ε*) are displayed in Table 1.

At all pHs, the pseudo-second-order model exhibited higher *R*^2^ values than the pseudo-first-order model. Compared with the predicted values by the pseudo-first-order model, the adsorption capacities predicted by the pseudo-second-order model were 160.97, 160.87, and 158.53 mg/g at pH 2.0, 5.0, and 7.0, respectively, which were close to the experimental *q_exp_* values (155.27, 153.30, and 153.26 mg/g at pH 2.0, 5.0, and 7.0, respectively). Moreover, the mean percentage error (*ε*) of the pseudo-second-order model was less than 5%, which was lower than that of the pseudo-first-order model. Therefore, the pseudo-second-order model was more suitable for depicting the kinetic experimental data from our experiments than the pseudo-first-order model, which illustrates that the adsorption rate of PEI/PVC-CF for RY2 may be controlled by chemisorption [31]. As shown in Table 1, the values of *k*_2_ and *h* decreased with an increasing pH value. These results show that, in the adsorption of RY2 by PEI/PVC-CF, the adsorption rate is affected by the pH of the dye solution and the adsorption rate decreases as the pH increases. Although the adsorption rate tended to decrease slightly as the pH increased, the dye uptake at equilibrium did not differ significantly at all pH conditions. This indicates that the optimized PEI/PVC-CF can be applied to wastewater treatment containing low concentrations of dyes over a wide pH range.

### 2.5. Adsorption Isotherms

**Figure 6:**
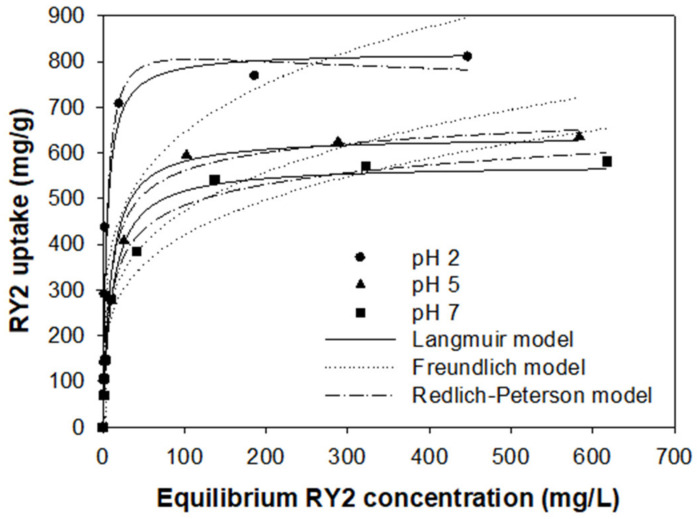
Adsorption isotherms of RY2 on the PEI/PVC-CF at different pH values (experimental conditions: *m* = 0.02 g, *V* = 30 mL, *C_i_* range = 100–1000 mg/L, and temperature = 25 °C).

Isothermal experiments were performed at pH 2.0, 5.0, and 7.0 to evaluate the maximum dye uptake of the optimized PEI/PVC-CF, and the results are displayed in Figure 6. The dye uptake in the adsorption equilibrium was dependent on the pH of the dye solution, and the amount of adsorption tended to decrease as the pH value increased. Typical adsorption isothermal curves were observed at all pH values evaluated. The isotherms were very close to the L-isotherm shape, which means that as the RY2 concentration increased, the ratio between the concentration of RY2 remaining in solution and adsorbed on the adsorbent decreased, giving a concave curve with a strict asymptotic plateau [32]. PEI/PVC-CF exhibited extremely high adsorption amounts for RY2, and the experimental dye uptake (*q_exp_*) at the plateau portion was 810.4, 635.8, and 580.8 mg/g at pH 2.0, 5.0, and 7.0, respectively. The slopes of the isotherms were also steep, so a high affinity of PEI/PVC-CF for RY2 can be expected.

Adsorption isotherm can provide information such as maximum adsorption capacity, adsorbate affinity, and favorability of the adsorption process [33]. To fully understand the adsorption isotherms, two- (Langmuir and Freundlich) and three-parameter (Redlich–Peterson) models were applied to depict the experimental data. The three isotherm models are represented as follows in Equations (4)–(6).

Langmuir model:

(4) $$q_{e}=\frac{q_{max}K_{L}C_{e}}{1+K_{L}C_{e}}$$

Freundlich model:

(5) $$q_{e}=K_{F}C_{e}^{1/n}$$

Redlich–Peterson model:

(6) $$q_{e}=\frac{K_{RP}C_{e}}{1+\alpha C_{e}^{\beta }}$$

where *q_e_* (mg/g) and *C_e_* (mg/L) represent the adsorption capacity and concentration of adsorbate at the equilibrium, respectiely, *q*_max_ (mg/g) is the maximum adsorption capacity, and *K_L_* (L/mg) is the Langmuir constant indicating the affinity between the adsorbent and adsorbate. *K_F_* (mg/g) is Freundlich constant related to adsorption capacity, and 1/*n* is a measure of surface heterogeneity, becoming more heterogeneous as the value approaches zero [34]. *K_RP_* (L/g) and *α* (L/mg)*^β^* are the Redlich–Peterson constants and *β* is the exponent, which lies between 0 and 1. When *β* = 1, the Redlich–Peterson model is reduced to the Langmuir model, while it is reduced to the Freundlich model when *β* = 0. If Henry’s law is suitable for all concentrations, *α* should be zero [35].

The model parameters calculated through the non-linear regression analysis by SigmaPlot 10.0 software are summarized in Table 2. As shown in Table 2, all adsorption isotherms fitted by the Langmuir model showed higher *R*^2^ values (0.950–0.990) than those of the Freundlich model (0.809–0.939). In addition, the *ε* values for the Langmuir model were less than 1.3%, which were much smaller than those for the Freundlich model (above 6.2%). This indicates that the Langmuir model is better suited to explain the adsorption isotherms of RY2 by PEI/PVC-CF. Since the *β* values of the Redlich–Peterson model are close to one, it further supports that the Langmuir model is more suitable to describe the isothermal adsorption results. The Langmuir equilibrium constant *K_L_* decreased with the increase of pH, indicating that the affinity between the adsorbent and adsorbate declined with the increase of pH. This may be the result of the deprotonation of the amine groups on the adsorbent, as mentioned in Section 3.3. The favorability of the adsorption process can be assessed by the dimensionless separation factor (*R_L_*), an essential characteristic of the Langmuir model as defined by Webber and Chakravorti. *R_L_* represents the type of the isotherm as either irreversible (*R_L_* = 0), favorable (0 < *R_L_* <1), liner (*R_L_* = 1), or unfavorable (*R_L_* > 1). The calculated *R_L_* values were in the range of 0.004 to 0.183, demonstrating that the RY2 adsorption process by PEI/PVC-CF was favorable.

In order to find out the level of the maximum dye uptake, the *q_max_* value of PEI-PVC-CF estimated from the Langmuir model was compared with the *q_max_* values of various adsorbents reported in the literature, and the results are summarized in Table 3. The *q_max_* value of PEI/PVC-CF was 820.6 mg/g, which is 2.4–7.0 times higher than other biosorbents such as *Corynebacterium glutamicum* biomass [14], polyurethane-immobilized *C. glutamicum* biomass [36], *Escherichia coli* biomass [37], esterified *E. coli* biomass [37], and dried activated sludge [38], 2.5 times higher than aminated mesoporous silica nanofiber [39], and 20.3 times higher than ion-exchange resin Amberjet 4200 [37]. In conclusion, the maximum dye uptake of PEI/PVC-CF is much higher than that of other sorbents, indicating that PEI/PVC-CF could be a promising adsorbent for RY2 removal in aqueous solutions.

### 2.6. Thermodynamic Analysis

**Figure 7:**
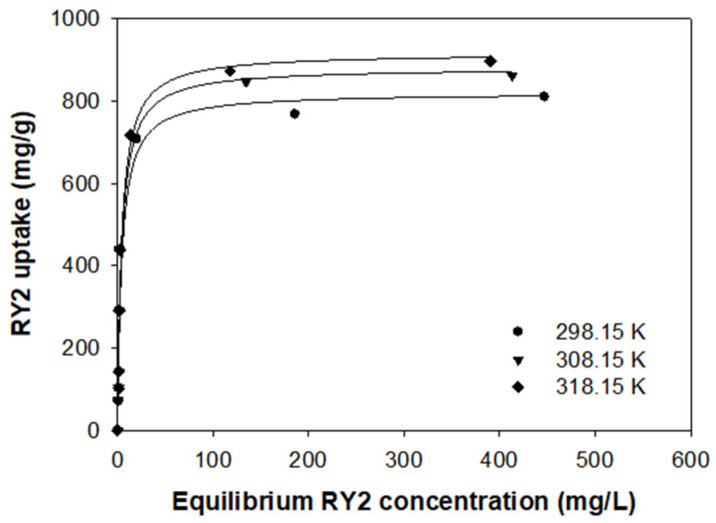
Adsorption isotherms of RY2 on the PEI/PVC-CF at different temperatures (experimental conditions: *m* = 0.02 g, *V* = 30 mL, *C_i_* range = 100–1000 mg/L, and pH = 2.0).

For the designing of an adsorption process, both enthalpy and entropy are vital factors that should be considered [40]. The change of thermodynamic parameters must be clarified to assess the feasibility and endothermicity of the adsorption process. Additional isotherm experiments were carried out at different temperatures (298.15, 308.15, and 318.15 K) to confirm the thermodynamic properties of PEI/PVC-CF for RY2 adsorption. As shown in Figure 7, the temperature change affected the adsorption amount and initial slope of the adsorbent. In particular, according to the Langmuir model, the maximum dye uptake of PEI/PVC-CF improved from 820.6 to 916.7 mg/g when the temperature increased from 298.15 to 318.15 K.

To evaluate the thermodynamic parameters, the change in Gibb’s free energy (*ΔG°*), enthalpy (*ΔH°*), and entropy (*ΔS°*) of the adsorption was estimated by the following Equations (7) and (8) [41].

(7) $$\Delta G{}^{\circ}=-RT\ln K_{d}$$

The van’t Hoff equation was applied to calculate the values of *ΔH°* and *ΔS°*:

(8) $$\ln K_{d}=\frac{\Delta S{}^{\circ}}{R}-\frac{\Delta H{}^{\circ}}{RT}$$

where *R* represents the gas constant (8.314 J/mol K), *T* stands for absolute temperature (K), and *K_d_* is the equilibrium constant at temperature *T*. The equilibrium constant *K* obtained from the best-fit isotherm model is not a dimensionless parameter that can be used directly for thermodynamic calculations, so it needs to be converted to a dimensionless parameter, as reported by Lima et al. [42]. The values of *ΔH°* and *ΔS°* were calculated by plotting ln*K_d_* versus 1/*T* of the linearized van’t Hoff equation. The thermodynamic parameters are displayed in Table 4. The value of Gibb’s free energy can indicate whether the adsorption process is spontaneous and favorable. At all studied temperatures, the *ΔG°* values were negative, indicating that the adsorption of RY2 on PEI/PVC-CF was spontaneous and favorable [43]. The *ΔG°* value decreased with the increase of temperature from 298.15 to 318.15 K, which revealed that the adsorption amount of RY2 increased with increasing temperature. The positive value of *ΔH°* intimated that the adsorption process was endothermic. The *ΔS°* value was above zero, which implied good affinity between RY2 and PEI/PVC-CF, as well as an increase of randomness at the solid-solution interface [44].

### 2.7. Desorption, Regeneration, and Reusability

**Figure 8:**
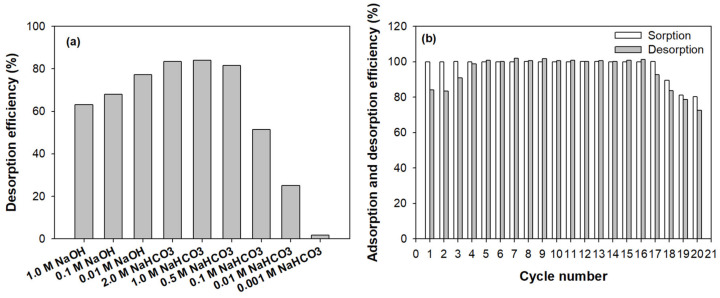
Screening for selecting the best concentration of eluent (**a**) and repeated adsorption–desorption cycles to evaluate the reusability of PEI/PVC-CF for RY2 (**b**).

The reusability of adsorbents is one of the important properties required for promising adsorbents. If exhausted adsorbents can be easily regenerated and reused, the operation cost of the treatment process and the disposal burden of adsorbents should be reduced. NaOH and NaHCO_3_ solutions were selected as eluents and investigated at various concentrations to find the best concentration for dye desorption. As shown in Figure 8a, the desorption efficiency of RY2 declined from 77.2% to 63.1% as the concentration of NaOH increased from 0.01 to 1.0 M. Conversely, as the concentration of NaHCO_3_ increased from 0.001 to 0.5 M, the desorption efficiency of RY2 increased sharply from 1.7% to 81.6%. As the concentration of NaHCO_3_ further increased to 2.0 M, the additionally desorbed dye from the adsorbent was only about 3.0%. Since the desorption efficiencies of 1.0 and 2.0 M NaHCO_3_ were almost the same at 83.5% and 84.0%, respectively, the 1.0 M NaHCO_3_ solution was chosen as the best eluent for reusability studies.

In order to evaluate the reusability of PEI/PVC-CF for RY2, a total of 20 cycles of adsorption–desorption experiments were repeatedly performed, and the results are displayed in Figure 8b. As shown in Figure 8b, the adsorption efficiency of PEI/PVC-CF to RY2 remained close to 100% until the 17th cycle, then gradually decreased, and showed an adsorption efficiency of 80.3% in the 20th cycle. On the other hand, desorption exhibited a different pattern from adsorption for a total of 20 cycles. In the first two cycles, the desorption efficiency kept at around 84.0%, then increased to 90.8%, and nearly reached 100% in the third and fourth cycles. After that, the desorption efficiency was maintained until the 16th cycle and gradually decreased again from the 17th cycle. Therefore, these results indicate that the optimized PEI/PVC-CF is a highly reusable adsorbent for dye treatment.

## 3. Materials and Methods

### 3.1. Materials

PVC (average molar weight 80,000) and *N,N*-dimethylformamide (DMF, 99.8%) were purchased from Sigma-Aldrich Korea Ltd. (Yongin, Korea) and Daejung Chemicals & Metals Co., Ltd. (Siheung, Korea), respectively. Branched PEI (M_W_ 70,000, content 50%) was purchased from Habjung Moolsan Co., Ltd. (Seoul, Korea). To avoid the PVC hardening by the water in PEI, the PEI was dried at 60 °C for 24 h in a drying oven before use. Sodium hydroxide, hydrogen chloride, and sodium hydrogen carbonate (NaHCO_3_) were supplied by Daejung Chemical Co. Ltd. (Siheung, Korea). A typical azo dye, RY2, was selected as a model dye and obtained from Sigma-Aldrich Korea. The general characteristics of RY2 are summarized in Table 5. All the other reagents used in this work were of analytical grade.

### 3.2. Preparation of PEI/PVC-CFs

In order to find the optimal conditions for fabricating a high-efficiency adsorbent for RY2, three different mass ratios (0.5:1.0, 1.0:1.0, and 1.0:1.25) of PEI and PVC and four different cross-linking times (4, 6, 8, and 12 h) were investigated. Several kinds of PEI/PVC-CFs were prepared on the basis of our previously reported method [45]. First, each sample of PVC and PEI was dissolved in 15 mL of DMF solution at 40 °C for 24 h. Then, the well-dissolved PVC and PEI solutions were mixed under predetermined mass ratios and agitated at 80 °C to induce cross-linking between the PEI and PVC molecules. After a certain cross-linking time (4, 6, 8 and 12 h), the PEI and PVC cross-linked polymer solution rapidly cooled down to room temperature. Thereafter, the synthesized polymer solution was wet-spun in distilled water through a needle with a 0.57 mm diameter to make fibrous PEI/PVC-CFs. The adsorbents produced under different fabricating conditions were washed several times with distilled water and freeze-dried for 24 h in a freeze dryer (TFD Series, Ilshinbiobase, Korea). Finally, the dried adsorbents were stored in a desiccator before use in the experiment.

### 3.3. Analytical Methods

Fourier transform infrared spectroscopy (FTIR) was used to confirm the presence of PEI and PVC in the PEI/PVC-CF using FT/IR-300E (Jasco, Japan). The infrared spectrum was obtained by the KBr (Potassium bromide) pellet method in the wavenumber range of 4000–400 cm^−1^. The surface morphology of PEI/PVC-CF was observed at magnifications of 100×, 300×, and 3000× using scanning electron microscopy (SEM, JSM-6400, Jeol, Japan). The Brunauer–Emmett–Teller (BET) surface area analysis of PEI/PVC-CF was performed on the micromeritics ASAP-2020M analyzer via N_2_ adsorption–desorption isotherms. The Barret–Joyner–Halenda (BJH) method was used to calculate the average pore diameter.

### 3.4. Adsorption Experiments

The RY2 stock solution (1000 mg/L) was prepared by dissolving RY2 in deionized water. In all batch adsorption experiments, 30 mL of RY2 solution and 0.02 g of PEI/PVC-CF were added to each 50-mL polypropylene conical tube. The tubes were placed in a shaking incubator and shaken at 25 °C and 160 rpm. The desired pH values were adjusted with 1.0 M HCl or 1.0 M NaOH solution. The adsorption capacities of PEI/PVC-CFs prepared under various manufacturing conditions were tested at pH 2.0 with an initial dye concentration of 500 mg/L. The effect of pH on the adsorption of RY2 by PEI/PVC-CF was carried out in the pH range of 2.0–12.0 for initial concentrations of 100 and 1000 mg/L, respectively. For adsorption kinetics, the experiments were conducted at different pH values (2.0, 5.0, and 7.0) using 100 mg/L of the initial RY2 solution. In the isotherm adsorption studies, the initial concentration of RY2 was varied from 50 to 1000 mg/L, and the experiments were carried out at pH 2.0, 5.0, and 7.0, respectively. Additional isotherm experiments were also performed under various temperatures (298.15, 308.15, and 318.15 K) to obtain thermodynamic parameters. After reaching the adsorption equilibrium, samples were taken and centrifuged for 5 min at 9000 rpm. A certain amount of supernatant was taken out and diluted appropriately with deionized water. The residual RY2 concentration was then analyzed using a spectrophotometer (X-ma 3000, Human, Korea) at 404 nm, which is the maximum absorption wavelength (λ_max_) of RY2 dye. The dye uptake was finally calculated using the following mass balance Equation (9):

(9) $$q=\frac{C_{i}V_{i}-C_{f}V_{f}}{m}$$

where *q* is the dye uptake (mg/g), *C_i_* and *C_f_* are the initial and final dye concentrations (mg/L), respectively, *V_i_* and *V_f_* represent the initial and final solution volumes (L), respectively, and *m* is the mass of adsorbent (g).

### 3.5. Desorption, Regeneration, and Reuse Experiments

For the desorption and regeneration experiments, RY2-loaded PEI/PVC-CFs were rinsed with acidic deionized water adjusted to pH 2.0 to remove unadsorbed dye molecules present on the adsorbent surface. Then, the RY2-loaded adsorbents were put in 30 mL of eluent and stirred at 25 °C and 160 rpm for 24 h to ensure sufficient desorption of RY2. NaOH and NaHCO_3_ solutions were used as eluents, and the optimal desorption condition was derived through desorption experiments carried out at various eluent concentrations (0.01–1.0 M for NaOH, 0.001–2.0 M for NaHCO_3_). Also, the adsorption–desorption experiment was repeated up to 20 cycles to evaluate the reusability of the adsorbent. The desorbed RY2 concentration was analyzed with a spectrophotometer and the desorption efficiency was calculated using the following Equation (10).

(10) $$\mathrm{Desorption}\;\mathrm{efficiency}\;\left(\%\right)=\;\frac{\mathrm{Desorbed}\;\mathrm{dye}\;\mathrm{weight}\;\left(\mathrm{mg}\right)}{\mathrm{Adsorbed}\;\mathrm{dye}\;\mathrm{weight}\;\left(\mathrm{mg}\right)}\;\times 100$$

## 4. Conclusions

PEI/PVC-CF with remarkable adsorption performance for RY2 was prepared at PEI:PVC = 1.25:1 (mass ratio), 6 h (reaction time), and 80 °C (reaction temperature). The adsorption and desorption properties of PEI/PVC-CF on RY2 were investigated through a variety of batch experiments. As a result, the optimal pH for RY2 adsorption by PEI/PVC-CF was observed at pH 2.0. The kinetic and isothermal experimental data were well described by the pseudo-second-order and Langmuir models, respectively. The maximum dye uptake at pH 2.0 and 25 °C was predicted to be 820.6 mg/g by the Langmuir model. Thermodynamic studies indicated that the adsorption of RY2 onto PEI/PVC-CF was favorable, and the adsorption process was spontaneous and endothermic. The RY2-loaded PEI/PVC-CF was effectively regenerated using 1.0 M NaHCO_3_ solution, and the adsorbent maintained high adsorption and desorption efficiencies for up to 17 repeated adsorption–desorption cycles. Therefore, the optimized PEI/PVC-CF can be used as a highly effective and reusable adsorbent for the removal of reactive dyes from aqueous solutions.

## Figures and Tables

**Table 1:** The parameters of kinetic models for RY2 adsorption on PEI/PVC-CF at pH 2.0, 5.0, and 7.0.

pH		Pseudo-First-Order Kinetics				Pseudo-Second-Order Kinetics				
	*q_exp_* (mg/g)	*q*_1_ (mg/g)	*k*_1_ (L/min)	*R* ^2^	*ε* (%)	*q*_2_(mg/g)	*k*_2_∙10^−4^ (g/mg min)	*h* (mg/g min)	*R* ^2^	*ε* (%)
2	155.27	145.05	0.011	0.917	6.58	160.97	0.985	2.55	0.965	3.67
5	153.30	143.59	0.009	0.929	5.03	160.87	0.819	2.12	0.976	4.94
7	153.26	141.97	0.008	0.923	7.37	158.53	0.759	1.91	0.969	3.63

*q_exp_*: the experimental values; *q*_1_: the amount of dye; *k*_1_: the pseudo-first-order rate constant; *R*^2^: the coefficient of determination; *ε*: the mean percentage errors; *q*_2_: the amount of dye; *k*_2_: the pseudo-second-order rate constant; *h*: the initial adsorption rate.

**Table 2:** The parameters of the isotherm models for RY2 adsorption onto PEI/PVC-CF.

pH	Langmuir				Freundlich				Redlich-Peterson			
	*q_max_* (mg/g)	*K_L_* (L/mg)	*R* ^2^	*ε* (%)	*K_F_* (L/g)	1/*n*	*R* ^2^	*ε* (%)	*K_RP_* (L/g)	*α* (L/mg)	*β*	*R* ^2^
2	820.6	0.224	0.950	1.26	234.0	0.220	0.809	25.26	166.8	0.160	1.045	0.953
5	636.0	0.107	0.990	0.03	158.0	0.238	0.917	9.19	84.4	0.178	0.948	0.994
7	574.6	0.089	0.982	1.07	137.1	0.243	0.939	6.26	77.3	0.224	0.912	0.993

*q_max_*: the maximum adsorption capacity; *K_L_*: the Langmuir constant indicating the affinity between the adsorbent and adsorbate; *K_F_*: the Freundlich constant related to adsorption capacity; 1/*n*: is a measure of surface heterogeneity; *K_RP_* and *α*: the Redlich–Peterson constants; *β:* the exponent, which lies between 0 and 1.

**Table 3:** Comparison of the maximum adsorption capacity of RY2 on various sorbents.

Sorbent	*q_max_* (mg/g)	Condition	Ref.
*C. glutamicum* biomass	154.3	pH 2.0, 25 °C	[14]
polyurethane-immobilized *C. glutamicum*	116.5	pH 2.0, 25 °C	[36]
*E. coli* biomass	196.9	pH 3.0, 25 °C	[37]
Esterified *E. coli* biomass	335.2	pH 3.0, 25 °C	[37]
Amberjet 4200	40.4	pH 3.0, 25 °C	[37]
Dried activated sludge	333.3	pH 5.0, 25 °C	[38]
Aminated mesoporous silica nanofiber	371.7	pH 3.0, 30 °C	[39]
PEI/PVC-CF	820.6	pH 2.0, 25 °C	This work

**Table 4:** Thermodynamic parameters of the adsorption of RY2 by PEI/PVC-CF.

Temp. (K)	*ΔG°* (kJ/mol)	*ΔH°* (kJ/mol)	*ΔS°* (J/mol K)
298.15	−30.19		
308.15	−31.23	1.21	105.35
318.15	−32.29		

*ΔG°:* energy; *ΔH°*: enthalpy; *ΔS°*: entropy.

**Table 5:** General characteristics of Reactive Yellow 2.

**Color Index No.**	**18972**	**Molecular Structure**	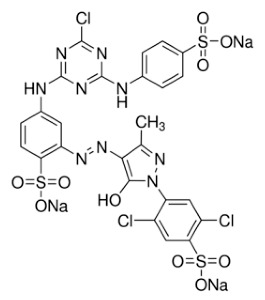
Molecular formula	C_25_H_15_Cl_3_N_9_Na_3_O_10_S_3_		
Molecular weight	872.97		
Dye content (%)	60–70		
*λ_max_* (nm)	404		

## Data Availability

Data set presented in this study is available in this article.

## Sample Availability

Samples of the compounds are available from the authors.
